# Effect of parathyroidectomy on epicardial fat thickness as a cardiovascular risk factor in patients with primary hyperparathyroidism

**DOI:** 10.3906/sag-1902-40

**Published:** 2019-08-08

**Authors:** Muhammed KIZILGÜL, Mustafa ÇALIŞKAN, Selvihan BEYSEL, Mustafa ÖZBEK, Erman ÇAKAL

**Affiliations:** 1 Department of Endocrinology and Metabolism, University of Health Sciences, Dışkapı Training and Research Hospital, Ankara Turkey

**Keywords:** Primary hyperparathyroidism, epicardial fat thickness, cardiometabolic risk factors

## Abstract

**Background/aim:**

Several studies demonstrated that primary hyperparathyroidism is related to increased risk for cardiovascular diseases (CVDs), and risk is decreased by parathyroidectomy. Epicardial fat thickness (EFT) has been postulated as a new marker of CVD risk. We evaluated the impact of parathyroidectomy on EFT in patients with primary hyperparathyroidism (PHPT).

**Materials and methods:**

Thirty-four PHPT patients (29 female, 5 male) and 28 age- and sex-matched controls (19 female, 9 male) were included in the study. Demographic, anthropometric, and biochemical data were recorded both before parathyroidectomy and 6 months after the procedure. Epicardial fat thickness was measured by transthoracic echocardiography.

**Results:**

Mean age was 53.15 ± 8.44 years. Mean preoperative EFT was higher than mean EFT in the control group (0.49 ± 0.07 cm to 0.46 ± 0.08 cm, P: 0.0005), and EFT decreased after parathyroidectomy (0.49 ± 0.07 cm to 0.44 ± 0.08 cm, P: 0.0005). Systolic blood pressure and calcium, parathormone, and hsCRP levels decreased after parathyroidectomy (P < 0.05). Vitamin D levels increased (P < 0.05). Diastolic blood pressure, body mass index, carotid intima-media thickness, and HOMA-IR, fasting plasma glucose, and phosphorus levels were unchanged after parathyroidectomy (P > 0.05). Preoperatively, EFT was correlated with SBP (r: 0.360, P: 0.0285) and age (r: 0.466, P: 0.0036). Multiple linear regression used to identify independent predictors of change in epicardial fat did not find any predictor of change in epicardial fat (P > 0.05).

**Conclusion:**

EFT was decreased by parathyroidectomy in patients with primary hyperparathyroidism.****However, the decrease in EFT was not correlated with any of the cardiovascular risk factors. More comprehensive studies evaluating the potential relation between PHPT and EFT need to be conducted.

## 1. Introduction 

Primary hyperparathyroidism (PHPT) is described as increased serum calcium and nonsuppressed parathyroid hormone (PTH) levels [1]. The incidence of PHPT is increasing significantly as laboratory screening tests become more widely available [2]. Accumulating data show that even mild PHPT leads to an elevated risk for cardiovascular diseases (CVDs), many of which improve after parathyroidectomy [3–7]. Epicardial fat (EF) is present between the outer layer of the myocardium and the inner layer of the pericardium. EF is metabolically active and may cause inflammation and dysfunction in the myocardium by secreting proatherogenic and proinflammatory cytokines, as well as reactive oxygen radicals. Increasing thickness of EF is related to higher risk of CVD [8]. Epicardial fat thickness (EFT) can be measured by transthoracic echocardiography [9] and is considered a novel marker of CVD risk [10]. We evaluated the impact of parathyroidectomy on EFT and its relation to cardiometabolic risk factors in patients with PHPT. 

## 2. Materials and methods

### 2.1. Patient selection

Thirty-four patients (29 female, 5 male) who were diagnosed with PHPT at the Dışkapı Training and Research Hospital between 2012 and 2016 and 28 age- and sex-matched controls (19 female, 9 male) were enrolled in the study. Local ethics committee approval was obtained, and all participants provided written informed consent before the study began. Patients with multiple endocrine neoplasias, parathyroid cancer, thyroid cancer, or hyperparathyroidism-jaw tumor syndrome and patients on drugs that counteract calcium and vitamin D metabolism were excluded from the study. A PHPT diagnosis was made in the case of persistent hypercalcemia with normal or nonsuppressed PTH concentrations. The decision for surgery was in agreement with guidelines applied at the time of diagnosis [11]. 

### 2.2. Clinical, biochemical, and hormonal measurements

Basal demographic data, clinical features, and carotid intima-media thickness (CIMT) measurements were recorded for all participants. Weight, height, waist circumference (WC), body mass index (BMI), and systolic and diastolic blood pressure (SDP and DBP, respectively) were measured. Fasting biochemical and hormonal measurements were taken in the morning using colorimetric methods, and complete blood counts were obtained from all participants. An intact chemiluminescent immunoassay of PTH (Immulite 2000) was used for measuring serum PTH levels, and 25-OH vitamin D concentrations were measured using a radioimmune assay. 

EFT was calculated using a Philips IE33, a commercially available device (Philips Electronics, USA), and was described as the distance between the visceral and parietal pericardium on the anterior wall of the right ventricle. EFT was calculated vertically on the free wall of the right ventricle in parasternal long- and short-axis views at end-diastole over 3 cardiac cycles [12]. Three measurements were performed and the average was recorded. High-resolution B-mode ultrasound (EUB 7000 HV; Hitachi, Japan) with a 13-MHz linear array transducer was used to image parathyroids. CIMT was measured by B-mode imaging high-resolution ultrasound (EUB 7000 HV; Hitachi).

### 2.3. Statistical analysis

All statistical analyses were performed using JMP 13.0.1 software (SAS Institute, USA). Descriptive data are reported as mean ± standard deviation (SD) and percentage (%). Normality of distribution was examined by using the Kolmogorov–Smirnov or Shapiro–Wilk W tests. The chi-square or Fisher’s exact test was used when variables were categorical. Student’s t-test was used for normally distributed continuous variables, and the Mann–Whitney U-test was used for those that did not fit a normal distribution. Correlations were assessed using Pearson’s and Spearman’s correlation. A multiple linear regression model was used to identify independent predictors of change in EF and P < 0.05 was accepted as statistical significance. 

## 3. Results

Thirty-four PHPT patients (29 female, 5 male) were included in the study. Mean age was 53.15 ± 8.44 years. The basal demographic, anthropometric, and biochemical parameters of patients are shown in Table 1. Preoperatively, EFT was correlated with SBP (r: 0.360, P: 0.0285) and age (r: 0.466, P: 0.0036) (Table 2). Mean preoperative EFT was higher than mean EFT in the control group (0.49 ± 0.07 cm to 0.46 ± 0.08 cm, P: 0.0005). Mean EFT decreased after parathyroidectomy (0.49 ± 0.07 cm to 0.44 ± 0.07 cm, P: 0.031). Systolic blood pressure and calcium, parathormone, and hsCRP levels decreased after parathyroidectomy (P < 0.05). Vitamin D levels increased (P < 0.05). Diastolic blood pressure, BMI, CIMT, and HOMA-IR, fasting plasma glucose, and phosphorus levels were unchanged after parathyroidectomy (P > 0.05) (Table 3). A multiple linear regression model was used to identify independent predictors of change in EF. We did not find a predictor of change in EF (Table 4).

**Table 1:** Clinical and demographic characteristics of patients.

	Mean ± SD
Age (years)	53.15 ± 8.44
BMI (kg/m2)	31.66 ± 7.71
Systolic blood pressure (mmHg)	141.03 ± 19.25
Diastolic blood pressure (mmHg)	86.38 ± 8.64
Calcium (mg/dL)	11.22 ± 0.55
Phosphorous (mg/dL)	2.83 ± 1.20
Parathormone (pg/mL)	226.97 ± 253.61
Vitamin D (ng/mL)	14.27 ± 15.40
Fasting plasma glucose (mg/dL)	93.50 ± 9.44
Creatinine (mg/dL)	0.73 ± 0.20
Albumin	4.54 ± 0.32
Alkaline phosphatase	119.00 ± 42.74
LDL-cholesterol (mg/dL)	126.33 ± 30.04
Triglyceride (mg/dL)	174.82 ± 88.56
HDL-cholesterol (mg/dL)	49.27 ± 12.48
CRP (mg/L)	3.13 ± 2.6
HOMA-IR	2.64 ± 1.50
CIMT (cm)	0.67 ± 0.12

**Table 2:** Correlation of EFT with metabolic and biochemical parameters.

	r2	P
Systolic blood pressure (mmHg)	0.3602	0.0285
Diastolic blood pressure (mmHg)	0.152	0.369
Adenoma volume (mL)	0.1768	0.325
Age (years)	0.4662	0.0036
Calcium (mg/dL)	–0.2057	0.222
Phosphorous (mg/dL)	0.0007	0.996
Parathormone (pg/mL)	–0.0252	0.882
Vitamin D (ng/mL)	–0.2014	0.232
Fasting plasma glucose (mg/dL)	0.3228	0.058
Creatinine (mg/dL)	0.1144	0.5
LDL-cholesterol (mg/dL)	–0.2013	0.239
Triglyceride (mg/dL)	–0.013	0.94
HDL-cholesterol (mg/dL)	0.1408	0.412
Waist circumference (cm)	0.3101	0.062
CRP (mg/L)	–0.0543	0.757
HOMA-IR	–0.0276	0.881
CIMT (cm)	0.0508	0.765
BMI (kg/m2)	0.3068	0.065

**Table 3:** EFT and metabolic parameters before and after parathyroidectomy.

Variables	Before parathyroidectomy (n: 33)	After parathyroidectomy(n: 33)	Control(n: 28)	P*	P**
Age (years)	53.15 ± 8.44		52.57 ± 6.03		0.755
EFT(cm)	0.49 ± 0.07	0.44 ± 0.08	0.46 ± 0.07	0.0005	0.031
SBP (mmHg)	140.68 ± 19.25	130.99 ± 17.11	122.31 ± 10.32	0.0026	<0001
DBP (mmHg)	86.96 ± 8.64	83.96 ± 8.64	78.95 ± 5.66	0.128	<0001
BMI (kg/m2)	31.76 ± 7.71	31.09 ± 4.88	29.87 ± 3.62	0.504	0.063
Ca (mmol/L)	11.21 ± 0.55	9.55 ± 0.58	9.39 ± 0.38	<0001	<0001
P (mmol/L)	2.82 ± 1.20	3.32 ± 0.60	3.45 ± 0.51	0.050	0.0017
PTH (pg/mL)	226.96 ± 253.61	64.89 ± 33.75	61.97 ± 25.25	0.0006	<0001
25(OH)D3 (ng/mL)	14.27 ± 15.39	28.97 ± 14.87	15.64 ± 12.07	0.0007	0.767
FPG (mmol/L)	93.50 ± 9.43	94.12 ± 14.08	88.3 ± 8.31	0.733	0.0021
hsCRP (mg/L)	3.13 ± 2.6	3.82 ± 2.16	3.23 ± 2.35	0.052	0.806
CIMT (cm)	0.66 ± 0.11	0.64 ± 0.11	0.61 ± 0.12	0.388	0.049
HOMA-IR	2.63 ± 1.49	2.43 ± 1.25	2.06 ± 1.03	0.256	0.041

*Before vs. after parathyroidectomy; data are represented as mean ± SD. **Before parathyroidectomy vs. control.

**Table 4:** Multiple linear regression model to identify independent predictor of change in epicardial fat.

	β	P
Sex	–0.152	0.489
Age	–0.296	0.180
Preoperative CRP	0.172	0.434
Adenoma size	–0.028	0.987
Preoperative PTH	–0.004	0.986
Preoperative calcium	–0.195	0.373
Preoperative BMI	0.058	0.792
Diastolic BP	–0.213	0.329
Systolic BP	–0.263	0.225
CIMT	–0.068	0.759

## 4. Discussion

Our aim was to evaluate the effect of parathyroidectomy on EFT and its relation to cardiovascular risk factors in PHPT patients. We found that EFT decreased after parathyroidectomy and was correlated with SBP and age. We believe that ours is the first study to evaluate the effect of parathyroidectomy on EFT in PHPT.

Adipose tissue is not just considered a primary site for fat storage; it also serves as an endocrine organ by secreting several hormones and adipokines including leptin, adiponectin, and tumor necrosis factor-α (TNF-α), which have proinflammatory, proatherogenic, or protective effects and lead to negative metabolic and cardiovascular outcomes [13,14]. Extraabdominal deposition of fat as epicardial and intraabdominal visceral adiposity are now suggested as markers of CVD risk [15–17]. Epicardial fat can synthesize and release adipokines and bioactive factors that may extend into the myocardium through vasocrine and/or paracrine pathways. Hence, EF tissue could be considered an endocrine organ [18]. As a result of functional and anatomical proximity, EF has a negative effect on coronary circulation and may lead to myocardial dysfunction and hypertrophy, and finally to coronary artery disease and cardiac insufficiency. EF is related to cardiometabolic disorders such as coronary artery disease, obesity, type 2 diabetes mellitus, and metabolic syndrome and with CVD risk factors such as hyperlipidemia, hypertension, obesity markers, and CIMT [8]. 

PTH receptors are present in cardiomyocytes, endothelial cells, and smooth muscle cells [19], and elevated PTH levels are related to myocardial fibrosis, calcification, and hypertrophy [20]. Additionally, elevated serum calcium levels are related to a higher rate of mortality, hypertension, left ventricular hypertrophy, and arrhythmias, as well as calcification of the myocardium, coronary arteries, and heart valves [21]. Studies have demonstrated that patients with PHPT had increased cardiovascular events and mortality, and many of these patients improved after parathyroidectomy [3–7,22]. Hypertension, hyperlipidemia, CIMT, CRP, and insulin resistance are all well-known CVD risk factors [23,24]. Numerous cardiovascular risk factors have been shown in patients with PHPT, including hypertension and elevated CIMT, insulin resistance, and CRP [25–28]. Asik et al. demonstrated that patients with PHPT had increased EFT. They also found correlations between EFT and CIMT, age, systolic blood pressure, and PTH and serum calcium levels [29]. In light of this information, we aimed to investigate whether EFT could be related to cardiometabolic risk factors and whether these decreased after parathyroidectomy in PHPT patients. In our study, EFT was correlated with some cardiometabolic risk factors such as SBP and age. Additionally, EFT decreased after parathyroidectomy in these patients. However, the decrease in EF thickness was not correlated with any of the cardiovascular risk factors.

Our study has some limitations since it was designed as a cross-sectional prospective study. Additionally, we had a relatively small sample size, and the study was conducted in a single center. We evaluated EF by 2-dimensional echocardiography. Although echocardiography is a relatively simple and cheap method, echocardiography might not fully represent the amount of EF tissue due to its 3-dimensional nature. This could be another limitation of our study.

As a result of the anatomical and functional proximity of EF tissue to coronary circulation, it can exert a direct effect on CVD risk. Our study showed that parathyroidectomy decreased the thickness of EF; however, this decrease was not correlated with any of the cardiovascular risk factors. More comprehensive studies evaluating the potential relation between PHPT and EFT need to be conducted.

## Acknowledgment

We would like to thank the cardiology doctors for performing echocardiography.
